# Supplementary material for: Reflections on contributing to health research: A qualitative interview study with research participants and patient advisors
Source: PLoS One. 2022 Dec 19;17(12):e0279354. doi: 10.1371/journal.pone.0279354 (PMC9762575; doi:10.1371/journal.pone.0279354)
Supplement: S1 File — (PDF) [file pone.0279354.s001.pdf]

Standards for Reporting Qualitative Research (SRQR)

No.	Topic	Item	Location in text
		**Title and abstract**	
**S1**	**Title**	Concise description of the nature and topic of the study. Recommend identifying the study as qualitative or indicating the approach (e.g. ethnography, grounded theory) or data collection methods (e.g. interview, focus group).	Title
**S2**	**Abstract**	Summary of key elements of the study using abstract format of the intended publication; typically includes background, purpose, methods results and conclusion.	Abstract, page 2
		**Introduction**	
**S3**	**Problem formulation**	Description of the significant of the problem / phenomenon studied; review of relevant theory and empirical work; problems statement.	Introduction, page 3-4
**S4**	**Purpose or research question**	Purpose of the study and specific objectives or questions.	Introduction, page 4
		**Methods**	
**S5**	**Qualitative approach and research paradigm**	Qualitative approach (e.g. ethnography, grounded theory, case study, phenomenology, narrative research) and guiding theory if appropriate; recommend identifying the research paradigm (e.g. postpositive, constructivist/interpretivist); rationale.*	Methods, study design, page 6
**S6**	**Research characteristics and reflexivity**	Researchers' characteristics that may influence the research, including personal attributes, qualifications / experience, relationship with participants, assumptions and / or presuppositions; potential or actual interaction between researchers' characteristics and the research questions, approach, methods, results, and / or transferability	Methods, research team and reflexivity, page 5 and methods, participants and recruitment, page 5
**S7**	**Context**	Setting / site and salient contextual factions; rationale	Methods, participants and recruitment, page 6 and acknowledgements
**S8**	**Sampling strategy**	How and why research participants, documents, or events were selected: criteria for deciding when no further sampling was necessary (e.g. sampling saturation): rationale.*	Table 1, page 6 and Methods, participants and recruitment, page 5
**S9**	**Ethical issues pertaining to human subjects**	Documentation of approval by an appropriate ethics review board and participant consent, or explanation if consent not gained; other confidentiality and data security issues	Methods, ethics approval and consent to participate
**S10**	**Data collection methods**	Types of data collected; details of data collection procedures including (as appropriate) start and stop dates of data collection and analysis, iterative process, triangulation of sources / methods, and modification of procedures in response to evolving study findings; rationale.*	Methods, study design, page 6 and Results, page 7
**S11**	**Data collection instruments and technologies**	Description of instruments (e.g. interview guides, questionnaires) and devices (e.g. audio recorders) used for data collection; if/how the instrument(s) changed over the source of the study.	Methods, study design, page 6 and reference 31
**S12**	**Units of study**	Number and relevant characteristics of participants, documents, or events included in the study; level of participation (could be reported in the results).	Table 2, page 8

**S13**	**Data processing**	Methods of processing data prior to and during analysis, including transcription, data entry, data management and security, verification of data integrity, data coding, and anonymisation / deidentification of excerpts.	Methods, study design, page 6
**S14**	**Data analysis**	Process by which inferences, themes etc., were identified and developed, including the researchers involved in data analysis; using references a specific paradigm or approach; rationale.*	Methods, study design, page 6
**S15**	**Techniques to enhance trustworthiness**	Techniques to enhance trustworthiness and credibility of data analysis (e.g. member checking, audit trail, triangulation); rationale.*	Methods, study design, page 6 and Results, page 7
		**Results / findings**	
**S16**	**Synthesis and interpretation**	Main findings (e.g. interpretations, inferences and themes); might include development of a theory or model, or integration with prior research or theory.	Results, page 8-22; Table 3 and Figure 1
**S17**	**Links to empirical data**	Evidence (e.g. quotes, field notes, text excerpts, photographs) to substantiate analytic findings.	Results, page 8-22 and Table 3
		**Discussion**	
**S18**	**Integration with prior work, implications, transferability, and contributions to the field**	Short summary of main findings; explanation of how findings and conclusions connect to, support, elaborate on, or challenge conclusions of earlier scholarship; discussion of the scope of application / generalisability; identification of unique contributions to scholarship in a discipline or field.	Discussion, page 23-28
**S19**	**Limitations**	Trustworthiness and limitation of findings.	Discussion, limitations, page 26-27

*The rationale should briefly discuss the justification for choosing that theory, approach, method, or technique rather than other options available, the assumptions and limitations implicit in those choices, and how those choices influence study conclusions and transferability. As appropriate, the rationale for several items might be discussed together.

Adapted from: O'Brien BC, Harris IB, Beckman TJ, Reed DA, Cook DA. Standards for reporting qualitative research: A synthesis of recommendations. Acad Med. 2014;89: 1245–1251. doi:10.1097/ACM.0000000000000388
