# Supplementary material for: Reflections on contributing to health research: A qualitative interview study with research participants and patient advisors
Source: PLoS One. 2022 Dec 19;17(12):e0279354. doi: 10.1371/journal.pone.0279354 (PMC9762575; doi:10.1371/journal.pone.0279354)
Supplement: Attachment — Submitted filename: ResponseToReviewers.docx [file pone.0279354.s002.docx]

Imperial College Healthcare NHS Trust

Imperial College London

London, UK

PLOS One

1 September 2022

Dear PLOS One Editorial Team,

Thank you to the editor and peer reviewers for your helpful feedback and recommendations. We have revised our manuscript as suggested and have resubmitted clean (Manuscript) and track-changed (Revised Manuscript with Track Changes) versions. We provide a point-by-point response in the table below, where the line numbers refer to the clean version of the manuscript.

To support the requested amendments, we have added the following additional references. No references were removed.

20.	Trusson D, Rowley E, Bramley L. A mixed-methods study of challenges and benefits of clinical academic careers for nurses, midwives and allied health professionals. BMJ Open. 2019;9: e030595. doi:10.1136/bmjopen-2019-030595

21.	Trusson D, Rowley E, Barratt J. Multimethods study comparing the experiences of medical clinical academics with nurses, midwives and allied health professionals pursuing a clinical academic career. BMJ Open. 2021;11: e043270. doi:10.1136/bmjopen-2020-043270

23.	Wenke R, Mickan S. The role and impact of research positions within health care settings in allied health: A systematic review. BMC Health Serv Res. 2016;16: 355. doi:10.1186/s12913-016-1606-0

24.	Black AT, Ali S, Baumbusch J, McNamee K, Mackay M. Practice-based nursing research: Evaluation of clinical and professional impacts from a research training programme. J Clin Nurs. 2019;28: 2681–2687. doi:10.1111/jocn.14861

25.	Wright D, Fry M, Adams J, Bowen C. Building research capacity in musculoskeletal health: qualitative evaluation of a graduate nurse and allied health professional internship programme. BMC Health Serv Res. 2020;20: 751. doi:10.1186/s12913-020-05628-1

26.	Sanders J, Malcolmson J, Philpott Jones S, Kelly J. Embedding post‐doctoral clinical academic careers in practice: The St Bartholomew’s Hospital model. J Clin Nurs. 2021. doi:10.1111/jocn.15575

29.	National Institute for Health and Care Research. Briefing notes for researchers - public involvement in NHS, health and social care research. 2021 [cited 15 Aug 2022]. Available: https://www.nihr.ac.uk/documents/briefing-notes-for-researchers-public-involvement-in-nhs-health-and-social-care-research/27371

37.	Wong G, Greenhalgh T, Westhorp G, Pawson R. Realist methods in medical education research: What are they and what can they contribute? Med Educ. 2012;46: 89–96. doi:10.1111/j.1365-2923.2011.04045.x

41.	Braun V, Clarke V. Using thematic analysis in psychology. Qual Res Psychol. 2006;3: 77–101. doi:10.1191/1478088706qp063oa

42.	Braun V, Clarke V. Thematic Analysis: A Practical Guide. London, UK: SAGE Publications Ltd; 2022.

We are extremely grateful for the peer reviewers’ suggestions. Your feedback has helped us improve the quality and transparency of reporting in our manuscript and we have acknowledged this contribution within the Acknowledgements.

Many thanks and best wishes,

Dr Lisa Newington

Corresponding author

Imperial College London

[l.newington@imperial.ac.uk](mailto:lisa.newington@gsst.nhs.uk)

| **EDITOR’S COMMENT** | **AUTHOR RESPONSE** |
| --- | --- |
| 1. Please ensure that your manuscript meets PLOS ONE's style requirements, including those for file naming. The PLOS ONE style templates can be found at  <https://journals.plos.org/plosone/s/file?id=wjVg/PLOSOne_formatting_sample_main_body.pdf> and  <https://journals.plos.org/plosone/s/file?id=ba62/PLOSOne_formatting_sample_title_authors_affiliations.pdf> | We have amended the formatting both within the document and for the file names. |
| 2. You indicated that you had ethical approval for your study. Please clarify whether minors were involved in this study. If so, in your Methods section, please ensure you have also stated whether you obtained consent from parents or guardians of the minors included in the study or whether the research ethics committee or IRB specifically waived the need for their consent. | Thank you for highlighting this lack of clarity. The study was limited to adults. We have added this information to Table 1. |
| 3. Please provide additional details regarding participant consent. In the ethics statement in the Methods and online submission information, please ensure that you have specified what type you obtained (for instance, written or verbal, and if verbal, how it was documented and witnessed). If your study included minors, state whether you obtained consent from parents or guardians. If the need for consent was waived by the ethics committee, please include this information.  Once you have amended this/these statement(s) in the Methods section of the manuscript, please add the same text to the “Ethics Statement” field of the submission form (via “Edit Submission”).  For additional information about PLOS ONE ethical requirements for human subjects research, please refer to <http://journals.plos.org/plosone/s/submission-guidelines#loc-human-subjects-research>. | We have added additional text to specify *written* informed consent. The exclusion of those <18 years has been clarified in Table 1.  We have updated the information in the submissions portal. |
| 4. Thank you for stating the following financial disclosure:   "LN was funded by an NIHR Imperial Biomedical Research Centre (BRC) and Imperial Health Charity Postdoctoral Research Fellowship (RFPO2122_3).  RKS was funded by an NIHR Imperial BRC Pan-London Research Fellowship (ICHT-39).  PK was supported by Imperial Health Charity, MW and CMA were supported by NIHR Imperial BRC. "  Please state what role the funders took in the study.  If the funders had no role, please state: "The funders had no role in study design, data collection and analysis, decision to publish, or preparation of the manuscript."  If this statement is not correct you must amend it as needed.  Please include this amended Role of Funder statement in your cover letter; we will change the online submission form on your behalf. | The funders had no role in the design or conduct of this research and we have added the requested statement.  The funding statement has been modified to include infrastructure support.  The revised text is also included in the new cover letter. |
| 5. Please expand the acronym “BRC” (as indicated in your financial disclosure) so that it states the name of your funders in full. This information should be included in your cover letter; we will change the online submission form on your behalf. | We have written BRC in full for each usage. This has been included in the new cover letter. |
| 6. In your Data Availability statement, you have not specified where the minimal data set underlying the results described in your manuscript can be found. PLOS defines a study's minimal data set as the underlying data used to reach the conclusions drawn in the manuscript and any additional data required to replicate the reported study findings in their entirety. All PLOS journals require that the minimal data set be made fully available. For more information about our data policy, please see <http://journals.plos.org/plosone/s/data-availability>.  "Upon re-submitting your revised manuscript, please upload your study’s minimal underlying data set as either Supporting Information files or to a stable, public repository and include the relevant URLs, DOIs, or accession numbers within your revised cover letter. For a list of acceptable repositories, please see [http://journals.plos.org/plosone/s/data-availability#loc-recommended-repositories.](http://journals.plos.org/plosone/s/data-availability%23loc-recommended-repositories.%20) Any potentially identifying patient information must be fully anonymized.  Important: If there are ethical or legal restrictions to sharing your data publicly, please explain these restrictions in detail. Please see our guidelines for more information on what we consider unacceptable restrictions to publicly sharing data: <http://journals.plos.org/plosone/s/data-availability#loc-unacceptable-data-access-restrictions>. Note that it is not acceptable for the authors to be the sole named individuals responsible for ensuring data access.  We will update your Data Availability statement to reflect the information you provide in your cover letter. | Thank you for this information.  We have deposited the data in the ReShare repository of the UK Data Service. This includes transcripts and the data arranged by code. We have included a data access statement and a template data sharing agreement.  We have also included a visualisation of our arrangement of the codes into themes within the repository in open access format.  The updated data access statement is provided in the new cover letter. |
| 7. Please include captions for your Supporting Information files at the end of your manuscript, and update any in-text citations to match accordingly. Please see our Supporting Information guidelines for more information: <http://journals.plos.org/plosone/s/supporting-information>. | We have amended the name of the supporting file. This has been amended in the text and on the file name. We have added summary information at the end of the manuscript. |
| 8. Please review your reference list to ensure that it is complete and correct. If you have cited papers that have been retracted, please include the rationale for doing so in the manuscript text, or remove these references and replace them with relevant current references. Any changes to the reference list should be mentioned in the rebuttal letter that accompanies your revised manuscript. If you need to cite a retracted article, indicate the article’s retracted status in the References list and also include a citation and full reference for the retraction notice. | We have checked the reference list. Several references have been added following recommendations from the reviewers. None were removed. New references are listed at the start of this response letter. |

| **REVIEWER 1 COMMENTS** | **AUTHOR RESPONSE** | **LOCATION** |
| --- | --- | --- |
| Reviewer #1: This is a valuable, interesting and well-written piece of research which will be of interest to a broad range of readers. I enjoyed reviewing this manuscript and believe, with some minor adjustments, it would meet the criteria for publication. As such, I have included a few suggestions below to strengthen this manuscript.  Overall:  Some of the language in the paper should be clarified – ‘involvement in research’ or ‘research involvement’ means something specific in the context of health research (i.e. research being carried out ‘with’ or ‘by’ members of the public). You use this term throughout the paper to refer to both participation and actual involvement. When discussing both concepts together you should use another term. For example ‘contribution to research’ (i.e. ‘..interviewees reflections on their contribution to research’). | Thank you for your interest in our work and for your helpful feedback. We appreciate your contribution to improving the quality of our manuscript.  We have reviewed our use of the terms ‘research involvement’ and ‘involvement in research’ and have replaced with ‘contribution to research’ or similar. Research involvement has been used only in relation to patient and public involvement.  We have also updated the title to reflect these changes:  *Reflections on contributing to health research: a qualitative interview study with research participants and patient advisors* | Throughout |
| Introduction: Later in the paper you mention that the aim of the research was not to differentiate between the views of PPI members and research participants – while I understand your reasons for including both of these groups and collating their views, I do think it is important to be very clear about how these groups differ in the earlier stages of the manuscript. The NIHR provide concise, helpful definitions of the differences between research participation and research involvement and I suggest these should be explicitly referred to in the introduction section (see <https://www.nihr.ac.uk/documents/briefing-notes-for-researchers-public-involvement-in-nhs-health-and-social-care-research/27371>). | Thank you for highlighting this ambiguity. We have provided an additional description of research participation and research involvement and have referenced the recommended NIHR briefing notes. The following text has been added:  *The UK National Institute for Health Research (NIHR) terminology refers to research ‘participation’ as taking part in a study, for example being recruited to a clinical trial or interview study (29). Research ‘involvement’ is defined as research being carried out with or by members of the public (29). This includes as members of a study advisory group or being involved in data collection or analysis.* | Introduction  Page 4  Line 69-72 |
| Methods:  In the abstract, you mention that you used Thematic Analysis (TA) but have not cited Braun and Clarke within the main paper and only given brief details of the steps that were taken. Please add more clarification within the methods section, around the method and process of data analysis. It would also be of benefit to add some more detail detailing the PPI contribution in relation to this project. For example, within the ‘research team and reflexivity’ section you state a public advisor contributed to the analysis, yet at line 123-126 there is mention only of the four authors. Can you provide further detail regarding how your public advisor was involved in this process? | Thank you for flagging this omission. We have added an additional description of the analysis in the methods section and cited Braun and Clarke. The amended text is:  *Data were analysed using Thematic Analysis (41,42). Initial inductive coding was completed by LN, RKS and PK on four transcripts. The preliminary codes were discussed and refined with input from all authors. An operational coding tree was developed and applied to the remaining transcripts by the lead author. Additional codes were added and defined through discussion with all authors. The final coding tree is available from the Open Science Framework (40). Codes were categorised into initial themes and sub-themes, which were discussed and refined by the authors and through feedback from a patient and public advisor (FA). During development, the themes were reviewed to ensure that they represented both consistent and differing views among interviewees. Data for research participants and patient advisory group members was not handled separately during the analysis. However, the composition of the themes and sub-themes was explored and any differences in perspective based on interviewee demographics (including type of research contribution) was discussed within the theme.* | Methods  Study design  Page 7  Line 139  Methods  Study design  Page 7  Line 139-49 |
| It may also be worth keeping in mind that data saturation is a contested concept within certain types of TA (see Braun & Clarke, 2021). | Thank you for raising the issue of data saturation. The term is included in the recommendation qualitative research checklists (COREQ and SRQR) and was required in our submission for ethics approval; therefore we used within our text. We took a blended approach to data saturation, with the aim of collecting sufficient high quality interview data to answer the research questions. This was guided by the recommendations in Braun and Clarke 2022 book.  We have amended as follows:  *Recruitment continued until the research team agreed that data saturation had been achieved. This was operationalised using a blended definition: no new items identified during the interview, no new codes identified during initial coding, and agreement that sufficient data had been collected to adequately address the research aims (39). The feasible sample size was anticipated to be between 15-25 interviews.* | Methods  Participants…  Page 6  Line 120-23 |
| Results:  Great to see lots of supporting quotations throughout the paper.  The sub-theme title for 2.3: ‘There wasn’t the two-way flow’ could more clearly convey the content of the theme (e.g. ‘Improving communication/information sharing between teams’ or similar). | Thank you for acknowledging our use of illustrative quotes.  The sub-theme titles were taken verbatim from the interviews. We understand that without the context of the whole quote, the title ‘there wasn’t the two-way flow’ is not sufficiently specific. We have amended the sub-theme title to *‘communication is everything’* | Results 2. Connecting…  Page 17  Line 293 and throughout |
| Discussion:  This study appears to have two linked but distinct aims 1) to identify strategies to optimise research experience for members of the public and 2) to understand what research impacts are important to the public. In places it felt like these two aims were conflated somewhat e.g. line 520-524 – Whilst I agree that providing progress updates and feedback to public advisors/participants is crucial, I don’t think this can be described as a ‘research impact’ in itself – it is a strategy to improve research experience. Please more clearly summarise your findings in relation to these two separate aims.  Research impact (changes that come about from the research study itself e.g. clinical outcomes, changes to practice etc.) and impact of public involvement (changes that come about from the inclusion of the public in research e.g. increased confidence, more relevant and accessible research outcomes) are again slightly different things that often seemed to be slightly confused/conflated when reading the manuscript. It may help to briefly delineate these different ‘types’ of impact perhaps within the intro and/or discussion sections. | Thank you for this helpful suggestion. We have clarified the aims at the start of the discussion:  *The aims were to understand more about the experiences of contributing to health research and to uncover the types of impacts that are important for these individuals.*  We have also provided additional signposting throughout the discussion to illustrate whether experience or impact are being discussed:  Perceived benefit of – replaced with *perceived beneficial impact*  We have added additional text to separate the discussion of personal impact with those at a broader population-focused level:  *The personal impacts that were identified when discussing research experiences did contribute to recommendations for impact capture, although interviewees largely focused on impacts at a broader organisational or societal level.* | Discussion  Page 24  Line 465-67  Page 26  Line 513  Page 26  Line 536-38 |
| Line 545-546 – you frame this as a limitation but I would not particularly consider it as such? I think it is a good point to acknowledge, but readers of this study will come from a range of backgrounds and/or interested in public views of impact more generally. For these reasons I think these findings have implications beyond NMAHPP research settings so can also be considered a strength of the study. | Thank you for this suggestion. We have added an additional sentence to emphasise the potential applicability to other settings:  *The issues and suggestions that were raised by interviewees were not unique to research led by NMAHPPs or research within these clinical areas. This is an unplanned strength of the current study, and as such, we hope that the findings will be useful for researchers beyond NMAHPP research settings.* | Discussion  Limitation  Page 28  Line 571-74 |
| Linked to my point above – I feel it would strengthen the discussion if you were to highlight how these findings could be useful/informative in the wider context (i.e. beyond just contributing to your framework). Public views around 1) improving the research experience and 2) important research impacts are valuable for health research more broadly! | Thank you for the encouragement to apply our findings to a broader setting. We have added the following text:  *Locally, these findings will be used to inform development of a framework to capture the impact of NMAHPP research. We also hope that the findings will be applied more broadly, with the aim of improving the research experience for participants and PPI groups, and emphasizing the types of research impact that are most meaningful to these contributors.* | Conclusion  Page 28  Line 584-87 |
| Within the conclusion you state ‘Routine sharing of relevant research data with the participants' clinicians was also recommended’ (line 553-554). I did not see adequate evidence of this within participants quotes and it isn’t clear whether you mean sharing individual level data (which may raise ethical concerns) or broader research findings. I feel it would be more accurate/relevant to either refine your wording or emphasise the value of information sharing in the broader context (i.e. to further facilitate translation of research findings into practice). | Thank you for highlighting this ambiguity. We have amended the text as follows:  *Routine sharing of relevant research data with clinicians was also recommended to aid the translation of research finding into practice.*  This has also been amended in the Abstract. | Conclusion  Page 28  Line 581-82  Abstract  Page 3  Line 41-2 |
| Ethical consideration:  It was good to see public involvement was included with the study design, materials development and analysis but I was slightly disappointed that public members were not named as part of the authorship team (although I can see they were mentioned in acknowledgements). If public members were involved in drafting/approving the manuscript, please consider whether they would like to be named as co-authors in line with IMCJE guidelines. If not, this would certainly be valuable to consider as you move forwards with your ongoing programme of work. | Thank you for these comments. The patient and public advisors for this project were not involved in writing and drafting the manuscript and therefore did not meet the requirements for authorship. However, you raise an important point and we will offer the opportunity for authorship for future manuscripts as part of this ongoing work. | N/A |

| **REVIEWER 2 COMMENTS** | **AUTHOR RESPONSE** | **LOCATION** |
| --- | --- | --- |
| Reviewer #2: Thank you for allowing me to review this interesting, insightful and well written manuscript. As a pharmacist researcher I found the narrative valuable for considering patient participant and PPIE needs/wants/requirements. The context for the study is adequately described with reference to appropriate literature from the NIHR. The findings are comprehensively reported, illustrated by a variety of quotes from across the sample. I found it particularly insightful to use short quotes as the 'names' for the sub-themes - this is something I have not seen before however ensures that the analysis is underpinned by the patient experience and true to the data. The discussion covers thoughtful topics of importance to NMAHPP researchers, such as the challenges we face with grants & resources and also provides some recommendations of how we can improve recruitment and retention of participants/ PPIE members, as well as make the experience of taking part in a study a more positive/ beneficial one. | Thank you for your interest and for this supportive feedback. | N/A |
| I only have a few minor comments/ questions for the authors as highlighted below: 1. Please check author's affiliations. I could not see who was affiliated with '3. Nursing Directorate'. | Thank you for raising this error. We have corrected the affiliations. MW was wrongly listed as 2,1 and has been corrected to 3.1. | Authors  Page 1  Line 10 |
| 2. Introduction, Pg. 3, line 56, 'demonstrate and evaluate the impact of this research involvement' - I was unsure what exactly the author's were referring to. Whose involvement? I am assuming PPIE/ patients but there has been no mention of them by this stage in the manuscript. | Thank you for highlighting this ambiguity. We have amended the text to remove the word involvement. We have also provided some additional information later in the introduction to clarify the definition of ‘participation’ and ‘involvement’ in response to reviewer 1. | Introduction  Page 4  Line 69-72 |
| 3. Introduction - It would be of value to describe why the focus on NMAHPP-led research, as opposed to all research (especially when research conducted by dentists & clinical doctors was excluded). What is it about NMAHPP research that is different, which has led to this piece of work? | Thank you for this suggestion. We have added the following additional contextual information in relation to NMAHPP research:  *However, in comparison to the medical professionals, there is currently a lack of structure and access to clinical academic career pathways for the NMAHPP disciplines (20–22). Various initiatives have been reported, such as embedded researcher models (23), practice-based research training programmes (24), interdisciplinary internship schemes (25), and organisation- and discipline-specific postdoctoral career pathways (26).* | Introduction  Page 3  Line 55-59 |
| 4. Introduction, Pg. 3, line 63 should 'researcher' be plural? | Thank you for identifying this typo. We have amended to ‘researchers’. | Introduction  Page 4  Line 68 |
| 5. Introduction, Pg. 4, lines 72-76 - I found this a rather large sentence. Suggest breaking up to improve readability if possible. Maybe at 'however'? | Thank you for this suggestion. We have split the sentence as recommended. | Introduction  Page 4  Line 74, Line 77 |
| 6. Research team and reflexivity - Pg. 5. I found this section to be lacking in any reflexivity. The backgrounds of the team were presented and their experience alluded to, however the section is missing what the impacts of this are on the study's conduct/ how this has influenced you. I also wondered if having completed your other work with clinicians might have given you some a priori insight and whether this influenced your data collection/ analysis? Did any personal/ professional beliefs incidentally affect this research? (S6 of SRQR checklist - need to incorporate some narrative around how these factors may have influenced the research/ researcher) | Thank you for the opportunity to provide more information about research team and our relationship with data collection and analysis. We have added the following text:  *The core research team had previously systematically reviewed NMAHPP research impact (27) and interviewed healthcare managers and research-active NMAHPPs about their experiences of research (28). Coding and analysis in the current study was not based on the findings from this previous work: we used an inductive coding approach with no preestablished coding ideas; and the initial coding and final discussions around themes and subthemes were supported by individuals not involved previously (RKS, PK, FA). However, we acknowledge that our own research experiences and awareness of the existing literature will have framed our approach.* | Methods  Research team…  Page 5  Line 104-10 |
| 7. Materials and Methods - was any theoretical lens/ perspective used to inform your study? (S5 of SRQR checklist; cannot identify within narrative) | We have added the following:  *Contextually, this research was situated within a realist perspective (37).* | Methods  Research team…  Page 5  Lines 101-2 |
| 8. Study design, Pg. 6, line 114 - should 'or their involvement' be 'of their involvement'? | Thank you for identifying this typo. It has been corrected to ‘of’. We have changed the word ‘involvement’ to ‘contribution’ based on feedback from reviewer 1. | Methods  Study design  Page 7  Line 130 |
| 9. Study design, Pg. 6 - I would have valued some content around the methods of data analysis. From the abstract and limited detail it sounds as though code book thematic analysis was used, however a description of how the codes were managed into the final thematic map would be useful. It would also be useful to know if PPI data has handled separately to patient participant data. Within the findings differing perspectives were offered between these groups and I wondered how you managed this in the analysis. | Thank you for this recommendation. We have added additional description of the analysis process:  *Data were analysis using Thematic Analysis (33,34). Initial inductive coding was completed by LN, RKS and PK on four transcripts. The preliminary codes were discussed and refined with input from all authors. An operational coding tree was developed and applied to the remaining transcripts by the lead author. Additional codes were added and defined through discussion with the team. The final coding tree is available from the Open Science Framework (32). Codes were categorised into themes and sub-themes, which were discussed and refined following feedback from a patient and public advisor (FA). During development, the themes were reviewed to ensure that they represented both consistent and differing views among interviewees. Data for research participants and patient advisory group members was not handled separately during the analysis. However, the composition of the themes and sub-themes was explored and any differences in perspective based on interviewee demographics (including type of research contribution) was discussed within the theme.* | Methods  Study design  Page 7  Line 139-49 |
| 10. Discussion, Pg. 24, lines 463, 464. This sentence around funding doesn't quite make sense/ is hard to read. I understand what you are trying to say, but think the structure of the sentence isn't quite right. I also suggest that you make it more explicit how the challenges of funding/ backfill mean that it is difficult to feedback about changes in practice | Thank you for highlighting this lack of clarity. We have amended the wording as follows:  *Feedback about these later impacts might be more challenging for researchers because the main modes of funding NMAHPP research are short-term grants or fellowships (28). Once the study is complete, the researcher may no longer have access to participants’ contact details or be permitted to contact them. Furthermore, they may not have allocated time for research activities. We encountered both issues when exploring recruitment options for the current study (45).* | Discussion  Page 24  Line 485-89 |
| 11. Discussion, Pg. 25, line 486, should be 'within the context of...' not 'with' | Thank you for identifying this typo. We have corrected to ‘within’. | Discussion  Page 25  Line 509 |
| 12. Figure 1. I can see that the blue colour of theme 4 represents (I think) the convergence of the 3 themes, however I am unsure exactly what the arrows represent. Are they crucial to the interaction of the themes in the analysis? If so, I would suggest this is made clearer.  Overall, I believe that this is a valuable piece of work for the NMAHPP community and has useful recommendations for all researchers working with patients/ PPIE. It is very well written and I would recommend it for publication with minor corrections. Thank you once again for the opportunity to review such important work. | Thank you for this suggestion. We have revised the figure to remove the arrows and have reformatted the shapes to (hopefully) better represent the convergence of the three themes with the concept of impact capture.  Thank you for your helpful feedback and interest in our work. It is very much appreciated. | Figure 1 |

| **REVIEWER 3 COMMENTS** | **AUTHOR RESPONSE** | **LOCATION** |
| --- | --- | --- |
| Reviewer #3: This paper describes the findings of a qualitative research project investigating the experiences of patients and citizens who contributed to health research done by non-medical/dental health professionals (NMAHPPs). Its findings will inform a framework for evaluating the impact of NMAHPP research. The paper gives a comprehensive account of the study's design and results, supplying additional data to supplement the extracts that illustrate the findings. It is methodologically sound and will be an important addition to the literature on the value of involving people in health research. What is particularly noteworthy about this publication is its focus on NMAHPP health research specifically, an area that the authors acknowledge has yet to be as fully embraced by the relevant professionals as that in medicine or dentistry. Hopefully this project and its outputs will give future NMAHPP researchers confidence and a sound footing from which to plan their research with impact in mind. I look forward to seeing the forthcoming Framework. | Thank you for reviewing our manuscript and for your endorsement. It is very much appreciated. | N/A |
