# Supplementary material for: Reflections on contributing to health research: A qualitative interview study with research participants and patient advisors
Source: PLoS One. 2022 Dec 19;17(12):e0279354. doi: 10.1371/journal.pone.0279354 (PMC9762575; doi:10.1371/journal.pone.0279354)
Supplement: Attachment — Submitted filename: Response to reviewers.docx [file pone.0279354.s003.docx]

Imperial College Healthcare NHS Trust

Imperial College London

London, UK

PLOS One

24 October 2022

Dear PLOS One Editorial Team,

Thank you to the peer reviewers for your helpful feedback and recommendations. We have revised our manuscript as suggested and have resubmitted clean (Manuscript) and track-changed (Revised Manuscript with Track Changes) versions. We provide a point-by-point response in the table below, where the line numbers refer to the clean version of the manuscript.

To support the requested amendments, we have added the following additional references. No references were removed.

38. Wiltshire G, Ronkainen N. A realist approach to thematic analysis: making sense of qualitative data through experiential, inferential and dispositional themes. J Crit Realis. 2021;20: 159–180

55. Braun V, Clarke V. To saturate or not to saturate? Questioning data saturation as a useful concept for thematic analysis and sample-size rationales. Qual Res Sport Exerc Heal. 2021;13: 201–216.

56. Amin MEK, Nørgaard LS, Cavaco AM, Witry MJ, Hillman L, Cernasev A, et al. Establishing trustworthiness and authenticity in qualitative pharmacy research. Res Soc Adm Pharm. 2020;16: 1472–1482.

Many thanks and best wishes,

Dr Lisa Newington

Corresponding author

Imperial College London

[l.newington@imperial.ac.uk](mailto:lisa.newington@gsst.nhs.uk)

| **Reviewer comment** | **Authors’ response** | **Location** |
| --- | --- | --- |
| Reviewer #1: The authors have thoroughly addressed all comments and I am more than happy to recommend this article be accepted for publication. | Thank you for your time in reviewing our manuscript and recommendation for publication. | N/A |
| Reviewer #2: Thank you for the opportunity to review this manuscript again. The authors have mostly answered my questions and addressed my feedback. I have 3 further and final comments. | Thank you for taking the time to re-review our manuscript and for the helpful suggestions to improve the clarity of our reporting. Please find our point-by-point responses summarised below. | N/A |
| 1. You now describe your theoretical underpinning as 'realist'. Please could you briefly describe how this perspective has shaped your work? | Thank you for the opportunity to discuss the theory informing our work. We have added the following additional text:  *Through this paradigm, we sought to explain the phenomenon of research impact through the individual views and experiences of our interviewees, with the interpretation that these individual experiences also contribute to a broader picture. This included the exploration of three aspects: experiential elements (describing interviewees’ subjective experiences and views); interferential elements (what might be inferred from these experiences); and dispositional elements (attempting to theorise about the phenomenon) [38].*  Reference added:  Wiltshire G, Ronkainen N. A realist approach to thematic analysis: making sense of qualitative data through experiential, inferential and dispositional themes. J Crit Realis. 2021;20: 159–180 | Research team and reflexivity  Page 5  Line 102-07 |
| 2. What particular factors guided your sample size? The manuscript mentions that sample size was determined by what was feasible. Was there anything else? Does your sample size limit or affect the quality or authenticity (<https://doi.org/10.1016/j.sapharm.2020.02.005>) of your work in any way? A comment in the strengths/ limitations section would be advisable. | Thank you for highlighting this recent article.  Our sample size was determined by a combination of factors. This was an iterative process. The interviewer made a note of whether new information was being reported in the interviews. During coding and analysis, all authors discussed whether saturation had been achieved. We also aimed to find interviewees for each of the characteristics in our purposive sampling criteria. We were also driven by feasibility in order to suggest an approximate sample size as part when designing the protocol and applying for ethics approval.  We have added to following text to the limitations, and included two additional references which are listed below.  *The number of interviews that were conducted (n=21) was guided by a blended definition of data saturation; no new concepts identified during the last 2-3 interviews and no new codes identified during analysis. The sample size proposed in the study protocol and application for ethics approval was based on a feasible range with our available resources (n=15-25). There are ongoing discussions within the field of qualitative healthcare research regarding the assessment of sample size, including whether data saturation is an appropriate concept and how this might relate to the credibility of the findings [40,55]. We are confident that our sample size and approach maximised the trustworthiness and authenticity of our findings [56], while acknowledging the limited ethnic diversity, as outlined above.*  References added:  Braun V, Clarke V. To saturate or not to saturate? Questioning data saturation as a useful concept for thematic analysis and sample-size rationales. Qual Res Sport Exerc Heal. 2021;13: 201–216.  Amin MEK, Nørgaard LS, Cavaco AM, Witry MJ, Hillman L, Cernasev A, et al. Establishing trustworthiness and authenticity in qualitative pharmacy research. Res Soc Adm Pharm. 2020;16: 1472–1482. | Limitations  Page 28  Line 571-78 |
| 3. You have mentioned that the analysis method was Thematic Analysis. Please be aware that current thinking is that there are 3 principle approaches to TA - Code reliability, code book and reflexive (see <https://doi.org/10.1007/s11135-021-01182-y> and <https://doi.org/10.1080/2159676X.2019.1628806>). The way it is currently described it sounds like code reliability - please could you accurately describe the approach to TA. This approach may then have implications for sample size (see point 2) and theoretical perspective (see point 1).  I hope this review is helpful. | Thank you for this helpful comment. We realise that the description of our thematic analysis was not sufficiently detailed and have added the following additional text to illustrate that our approach was primarily reflexive rather than based on a code reliability or code book approach:  Data were analysed using *Reflexive* Thematic Analysis [42,43]. Initial inductive coding was completed by LN, RKS and PK on four transcripts. The preliminary codes were discussed and refined with input from all authors. An operational coding tree was developed and applied to the remaining transcripts by the lead author.  *This was not a rigid codebook, rather a method of keeping track of the coding thoughts and descriptions as the research team were all working on multiple different projects.* Additional codes were added and defined through discussion with all authors. *Codes were also redefined, relabelled, merged, and expanded as part of this iterative analysis process, which involved reviewing and recoding earlier interviews in light of changes to the coding content and structure.* | Study design  Page 7-8  Line 144-51 |
